# THE IMPACT OF DEMENTIA ON SLEEP QUALITY DURING COVID-19

**DOI:** 10.1093/geroni/igad104.3017

**Published:** 2023-12-21

**Authors:** Susanny Beltran, Talib Hakim, Zainab Suntai

**Affiliations:** University of Central Florida, Orlando, Florida, United States; University of Central Florida, Orlando, Florida, United States; Baylor University, Waco, Texas, United States

## Abstract

Sleep disturbances are documented among older adults, and it is well-known that COVID-19 disproportionately affected older adults. Yet, studies on sleep quality among older adults with dementia, specifically, tend to focus on caregiver sleep. The purpose of this study was to examine sleep quantity and quality among older adults during 2020. Data were taken from the 2020 National Health and Aging Trends study, an annual study of Medicare beneficiaries aged 65 and older. Chi-square tests were used for bivariate analysis, and multivariate logistic regression models were used to assess differences in sleep quantity and quality between dementia and non-dementia older adults in the national sample. Results revealed that during 2020, the year the COVID-19 pandemic appeared in the U.S., older adults with dementia reported less sleep, and lower quality of sleep compared to those without dementia. This study demonstrates that sleep disturbances are significant among dementia patients. Poor sleep has previously been linked to other risks affecting quality of life, such as falls and caregiver stress, and nonpharmacological approaches are preferred for dementia patients given risks associated with psychotropics. Thus, there is a need for further study to explore changes in sleep quality over time, and to develop non-pharmacological interventions to support dementia patients’ sleep quality and quantity.

